# Physiology and Molecular Response Mechanisms in the Gills of *Macrobrachium rosenbergii* Under Acute NaHCO_3_ Alkaline Stress

**DOI:** 10.3390/antiox14101266

**Published:** 2025-10-21

**Authors:** Heng Yu, Songbao Zou, Huwei Yuan, Mei Liu, Meng Ni, Julin Yuan

**Affiliations:** 1 Key Laboratory of Healthy Freshwater Aquaculture, Ministry of Agriculture and Rural Affairs, Key Laboratory of Fish Health and Nutrition of Zhejiang Province, Zhejiang Institute of Freshwater Fisheries, Huzhou 313000, China; yuheng166@126.com (H.Y.);; 2 College of Fisheries and Life Science, Shanghai Ocean University, Shanghai 201306, China

**Keywords:** NaHCO_3_, *Macrobrachium rosenbergii* (*M. rosenbergii*), gills, molecular response mechanisms, antioxidant and immune enzyme activity

## Abstract

As an important freshwater economic shrimp, *Macrobrachium rosenbergii* (*M. rosenbergii*) possesses a certain tolerance to saline/alkaline conditions. Analyzing the damage mechanism and stress response of *M. rosenbergii* in saline/alkaline environments will provide a scientific basis for promoting ecological restoration through the utilization of saline/alkaline water resources for aquaculture. In the first experiment, the 96 h median lethal concentration (LC_50_) of NaHCO_3_ was determined for juvenile *M. rosenbergii*. A second experiment then exposed the shrimp to a control group and an alkaline water group set at 60% of the established LC_50_. After 96 h of exposure, gill tissue samples were collected from both groups for analysis. The aim was to clarify both the damage mechanisms induced by NaHCO_3_ and the response mechanisms. The current results indicated that acute NaHCO_3_ exposure reduced antioxidant enzyme activity and induced gill tissue damage in *M. rosenbergii*. In response to the stress caused by NaHCO_3_, *M. rosenbergii* activated immune-related enzymes as well as immune-related differentially expressed genes involved in endocytosis, autophagy, and the toll-like receptor signaling pathway. In summary, the current research provided reference information for understanding the adverse effects caused by saline/alkaline water stress and for the breeding of *M. rosenbergii* in saline/alkaline water environments.

## 1. Introduction

In the context of global climate change, abiotic stresses such as strong winds, drought, soil salinization, and extreme temperatures (including heat stress and cold stress) pose major challenges to the growth of organisms. Among these, soil salinization is indeed a key environmental constraint that severely limits the functionality of agricultural soil ecosystems globally [1]. Once established, soil salinization constitutes a significant environmental challenge generating substantial adverse impacts, including primarily ecological degradation, loss of land productivity, exacerbated desertification, potential water contamination, and economic losses [2,3,4]. Saline/alkaline land resulting from soil salinization is an outcome of the combined influence of specific environmental conditions (aridity/scarce rainfall, saline groundwater) and human activities (primarily improper irrigation practices), representing a global ecological threat [5]. Saline/alkaline lands represent vital ‘dormant resources’ and highly valuable ‘potential granaries’. Particularly regarding saline/alkaline water bodies, they are extensively distributed worldwide, with China alone containing approximately 4.6 million hectares of such waters. The development of aquaculture in saline/alkaline lands can not only effectively expand fisheries’ production capacity to better meet public demand for premium aquatic products but also boost economic growth in affected regions. Crucially, it carries significant potential for mitigating water scarcity crises and rehabilitating degraded saline soil/water ecosystems. In China, the predominant types of saline/alkaline water are chloride-type, sulfate-type, and carbonate-type. Compared to chloride-type and sulfate-type waters, carbonate-type saline/alkaline water poses more severe detrimental effects on aquatic animals [6,7]. Carbonate alkalinity stress has been identified as one of the primary factors affecting the survival of aquatic animals in saline/alkaline aquatic environments. Previous studies have demonstrated that exposure to elevated carbonate concentrations induces significant alterations in biochemical parameters and histopathological damages of aral barbel (*Luciobarbus capito*) [8] and oriental river prawn (*Macrobrachium nipponense*) [9]. 

Selecting and cultivating dominant species with alkali-tolerant traits represent a pivotal strategy for optimizing the utilization of saline/alkaline water resources in aquaculture. Crustaceans exhibit varying degrees of carbonate alkalinity tolerance, as evidenced by species such as the pacific white shrimp (*Litopenaeus vannamei*) [10], ridgetail white prawn (*Exopalaemon carinicauda*) [11], and Chinese mitten crab (*Eriocheir sinensis*) [12]. As an economically important freshwater shrimp in China, the giant freshwater prawn (*Macrobrachium rosenbergii*) was introduced in 1976. After four decades of development, it has become a significant aquaculture species, and China’s *M. rosenbergii* production reached 196,374 tonnes in 2023. Possessing a certain level of tolerance, *M. rosenbergii* shows promise as an emerging species for cultivation in saline/alkaline waters. However, saline/alkaline water bodies, particularly alkaline environments characterized primarily by high carbonate alkalinity, impose multiple stressors on the physiological metabolism of aquatic animals. It has been reported in studies on shrimp and crabs that carbonate alkalinity stress could impair the organism’s antioxidant defense system, manifested as a reduction in antioxidant enzyme activities (such as superoxide dismutase (SOD), catalase (CAT), glutathione peroxidase (GSH-Px), and total antioxidant capacity (T-AOC)) [10,13], and even inducing oxidative damage in tissues. This leads to critical bottlenecks constraining industrial development, such as inhibited growth and decreased survival rates. Therefore, elucidating the damage mechanisms and molecular response mechanisms of *M. rosenbergii* under alkaline exposure was of considerable significance. Chen et al. [7] conducted transcriptomic sequencing on *M. rosenbergii* larvae exposed to carbonate/alkali stress. However, differences in size, morphology, and sampled tissues between larvae and juveniles may lead to divergent stress response mechanisms. Furthermore, pathological damage to *M. rosenbergii* tissues under carbonate/alkali stress remains poorly documented.

Gills are important respiratory organs of shrimps, changes in physiological indicators, histomorphological alterations, and pathological damage in gills can serve as critical evidence for assessing injuries induced by carbonate/alkali stress [14]. And gills play critical roles in defending against saline/alkaline stress conditions; when exposed to external environmental stimuli, crustaceans elicit stress responses characterized by an increase in immune-related enzyme activities (such as alkaline phosphatase (ALP), acid phosphatase (ACP), nitric oxide (NO), and nitric oxide synthase (T-NOS)) within a specific range, reflecting a protective mechanism activated by the organism [15]. In addition, the gill tissue molecules of crustaceans will also respond to cope with the carbonate alkalinity stress [16], and transcriptomics has emerged as a powerful tool for elucidating the molecular response mechanisms of crustaceans to carbonate/alkali stress [12,14]. Therefore, focusing on gill tissue and building upon our prior findings [17], the current research mainly focused on the morphological changes in gill tissues and the changes in the response mechanisms of enzyme activities and molecules in gill tissues of *M. rosenbergii* after 96 h of acute sodium bicarbonate (NaHCO_3_) alkali stress. The current study revealed the physiological mechanism by which *M. rosenbergii* responds to alkaline carbonate stress and the damage characteristics it causes. And it provided a crucial scientific basis for utilizing saline/alkaline water resources for shrimp farming, as well as for promoting the ecological restoration and environmental governance of saline/alkaline lands.

## 2. Materials and Methods

### 2.1. Ethics Statement

In the present study, the treatment method of *M. rosenbergii* strictly followed the Zhejiang Institute of Freshwater Fisheries Animal Experimentation Ethics Committee (ZJIFF20210302); approval date: 2 March 2021.

### 2.2. Experimental Design

For Experiment 1, a total of 360 healthy and similar juvenile *M. rosenbergii* (average weight of 1.30 ± 0.02 g and average body length of 3.50 ± 0.02 cm) were randomly selected and divided into 18 white plastic farming boxes (45 L), and 6 groups were set with 3 parallel in each group, and 20 shrimp in each parallel. They were temporarily raised in an exposed water environment for 7 days to adapt to the farming environment. According to the acute NaHCO_3_ alkali stress experiment conducted by Chen et al. on *M. rosenbergii* larvae (0.010 ± 0.001 g), a semi-lethal concentration (LC_50_) of NaHCO_3_ at 96 h was determined to be 5.69 mmol/L [7]. In the current study, tap water was used as the control group, and NaHCO_3_ was used to prepare alkaline water as the experimental group, with NaHCO_3_ gradients set at 10 mmol/L, 12 mmol/L, 14 mmol/L, 16 mmol/L, 18 mmol/L, and 20 mmol/L, and the formal experiment was conducted after NaHCO_3_ was fully dissolved and stabilized in the water for 24 h. We recorded the number of deaths of juvenile *M. rosenbergii* in each parallel after 24 h, 48 h, 72 h, and 96 h of acute stress on *M. rosenbergii* in NaHCO_3_ water.

For Experiment 2, a total of 150 healthy and similar juvenile *M. rosenbergii* (average weight of 1.29 ± 0.02 g and average body length of 3.46 ± 0.03 cm) were randomly selected and divided into 6 white plastic farming boxes (45 L), and 2 groups were set with 3 parallel in each group, and 25 shrimp in each parallel. The 60% concentration of the LC_50_ of NaHCO_3_ alkalinity (6.4 mmol/L) obtained after 96 h in Experiment 1 was taken as the experimental group, and tap water was used as the control group. During the above two experiments, the water temperature was 28 ± 1 °C, and the dissolved oxygen was not allowed to be lower than 6 mg/L.

### 2.3. Sample Collection

After 96 h of NaHCO_3_ alkalinity stress on *M. rosenbergii* in Experiment 2, the tissues of the *M. Rosenbergii* in the control group and the experimental group were collected. Firstly, the gill tissues of 6 shrimp (18 shrimp were sampled from the control group, and another 18 from the experimental group) were collected from each parallel for the determination of antioxidant and immune-related enzyme activities and the relative gene expression levels. Secondly, the gill tissues of 6 shrimp in each parallel (18 shrimp were sampled from the control group, and another 18 from the experimental group) were collected for transcriptomic analysis. To mitigate the effects of individual variation and limited sample size, we pooled gill tissues from 6 randomly selected shrimp per replicate to form a single composite sample for subsequent transcriptomic analysis. The collected gills were immediately frozen in liquid nitrogen and then transferred to a refrigerator at −80 °C for storage. Finally, the gill tissues of 1 shrimp (3 shrimp in each group) were collected from each parallel and placed in 4% paraformaldehyde and 2.5% glutaraldehyde, separately, for the detection of gill tissues and ultrastructure observation.

### 2.4. Histological Examination of Gills (HE)

Collected samples were fixed in 4% paraformaldehyde. After adequate fixation, the following procedures were performed: (1) Fixation and dewaxing: Tissue samples were paraffin-embedded using an embedding machine (JB-P5, Wuhan Junjie Electronics Co., Ltd., Wuhan, China), and sections were prepared using a cryostat (CRYOSTAR NX50, Thermo Fisher Scientific Co., Ltd., Shanghai, China); (2) Hematoxylin staining: Sections were stained with hematoxylin for 3–5 min and washed with tap water; (3) Eosin staining: Sections were dehydrated in 95% ethanol for 1 min and stained with eosin for 15 s; (4) Dehydration and mounting: Sections were sequentially dehydrated in anhydrous ethanol I (2 min), anhydrous ethanol II (2 min), anhydrous ethanol III (2 min), n-butanol I (2 min), n-butanol II (2 min), xylene I (2 min), and xylene II (2 min) for transparency, followed by neutral resin mounting; (5) Microscopy and imaging: Sections were examined under a bright-field microscope (NIKON ECLIPSE E100, Nikon Corporation, Tokyo, Japan), and images were captured and analyzed using an imaging system (NIKON DS-U3, Nikon, Japan).

### 2.5. Ultrastructure Observation

(1) Tissue fixation: Tissue fragments (1 mm^3^) were fixed in electron microscopy fixative (G1102, Wuhan Saiweier Biotechnology Co., Ltd., Wuhan, China), followed by three washes (15 min each) with 0.1 M phosphate buffer (PB, pH 7.4). (2) Dehydration: Tissues were dehydrated at room temperature in a graded ethanol series (30%, 50%, 70%, 80%, 95%, 100%, and 100%; 20 min each) and 100% acetone (twice, 15 min each). (3) Infiltration and embedding: Pure 812 embedding resin (product no. 90529-77-4, SPI) was poured into embedding molds. Samples were inserted into the molds and incubated overnight in a 37 °C oven. (4) Polymerization: Molds were transferred to a 60 °C oven for 48 h to polymerize the resin blocks. (5) Orientation: Resin blocks were sectioned using an ultramicrotome (Leica UC7, Leica Microsystems GmbH, Wetzlar, Germany). Semi-thin sections were stained with toluidine blue and examined under a light microscope to locate target regions. (6) Ultra-thin sectioning: Selected resin blocks were ultra-thin-sectioned using an ultramicrotome (Leica UC7), and sections were collected on 150-mesh copper grids. (7) Staining: Grids were stained with 2% uranyl acetate in ethanol (8 min, protected from light), rinsed three times with 70% ethanol, washed three times with ultrapure water, counterstained with 2.6% lead citrate (8 min), and rinsed three times with ultrapure water. Excess liquid was blotted with filter paper, and grids were air-dried overnight at room temperature. (8) Imaging: Sections were observed under a transmission electron microscope (HT7800/HT7700, Hitachi High-Technologies Corporation, Tokyo, Japan), and images were acquired for analysis. 

### 2.6. Determination of Antioxidant and Immune-Related Enzyme Activities

All antioxidant (superoxide dismutase (SOD), anti-superoxide anion free radical (ASAFR), glutathione peroxidase (GSH-Px), catalase (CAT), total antioxidant capacity (T-AOC), glutathione s-transferase (GST)), and immune indices (alkaline phosphatase (ALP), acid phosphatase (ACP), nitric oxide synthase (T-NOS), and nitric oxide (NO)) were measured using commercial assay kits (Nanjing Jiancheng Bioengineering Institute, Nanjing, China) according to the manufacturer’s protocols. Enzyme activities were quantified using specified instruments as per the kit instructions.

### 2.7. RNA Extraction, Library Preparation, and Library Quality Inspection

(1) RNA extraction: Total RNA was isolated from tissue samples using TRIzol reagent (Thermo Fisher Scientific Inc., Waltham, MA, USA). (2) Library preparation: Ribosomal RNA (rRNA) was depleted using a standard rRNA removal kit. Enriched mRNA was reverse-transcribed into double-stranded cDNA. cDNA ends were repaired, adapters were ligated, and libraries were amplified by PCR. Qualified libraries were subjected to sequencing. (3) RNA quality control: RNA integrity and DNA contamination were assessed by agarose gel electrophoresis (1.5% agarose in 1× TAE buffer(Thermo Fisher Scientific Inc., Waltham, MA, USA)). RNA purity was evaluated using a NanoPhotometer spectrophotometer (Implen GmbH, Munich, Germany). 

### 2.8. Quality Assessment of Transcriptome Sequencing Data

To ensure data quality, raw reads were subjected to preprocessing to eliminate interference from low-quality or irrelevant data prior to bioinformatic analysis. First, raw sequencing data were processed using fastp for quality control [18]. Low-quality reads were filtered based on the following criteria: (1) removal of reads containing adapter sequences; (2) exclusion of reads composed entirely of poly A sequences; (3) discarding reads with low-quality bases (Q ≤ 20) constituting > 50% of the read length. The resulting clean reads were subsequently aligned to a species-specific ribosomal RNA (rRNA) database to remove residual rRNA-derived sequences [19]. Unmapped reads were retained for transcriptomic analysis. Finally, for reference genome-based alignment, HISAT2 was employed to map clean reads to the reference genome. After StringTie-based transcript assembly, RSEM [20] was employed to quantify gene expression levels. Differential gene expression analysis was performed using DESeq2 [21], with significance thresholds defined as a false discovery rate (FDR) < 0.05 and |log2(fold change)| > log2(2). Genes meeting these criteria were identified as significantly differentially expressed.

### 2.9. Quantitative Real-Time PCR (qPCR)

Tissues were ground in liquid nitrogen, and total RNA was extracted using FreeZol Reagent (R711-01, Vazyme, Nanjing, China). qPCR was conducted using HiScript^®^ II One Step qRT-PCR SYBR Green Kit (Q221-01, Vazyme, Nanjing, China) on a 7500 Real-time PCR system (Thermo Fisher Scientific Inc., Waltham, MA, USA). Primers were designed based on transcriptome sequences and validated using Primer Premier 6 software, and the primers were synthesized by Sangon Biotech (Shanghai, China) (Table 1). Beta-actin (β-actin) was used as the housekeeping gene, and relative gene expression was normalized to the housekeeping gene and analyzed using the 2^−ΔΔCT^ method [22].

### 2.10. Statistical Analysis

All data were tested by the software SPSS 20.0 for homogeneity of variance, the Tukey method in one-way variance was used to compare data between multiple groups, and an independent sample T-test was used for the two groups of data (*p* < 0.05). The LC_50_ of 24 h, 48 h, 72 h, and 96 h alkalinity for juvenile *M. rosenbergii* was determined using SPSS 20.0 software, and the LC_50_ was calculated through Probit regression. The safe concentration was calculated by 48 h LC_50_ × 0.3/(24 h LC_50_/48 h LC_50_)^2^. The results were expressed as the mean ± standard error, with different superscript letters representing significant differences (*p* < 0.05).

## 3. Results

### 3.1. Determination of LC_50_ of NaHCO_3_ Alkalinity

**Figure 1:**
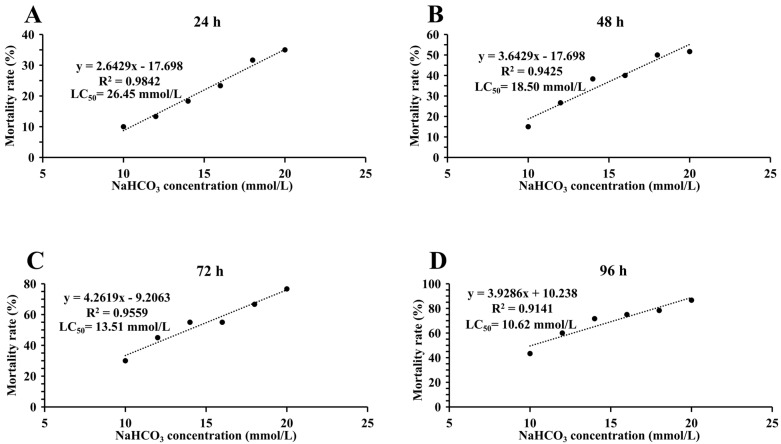
(**A**–**D**) The mortality rates of *M. rosenbergii* at different concentrations of NaHCO_3_ at 24 h, 48 h, 72 h, and 96 h, respectively.

As shown in Figure 1, with the increase in NaHCO_3_ alkalinity, the mortality rate of *M. rosenbergii* also gradually increased. The LC_50_ of NaHCO_3_ of *M. rosenbergii* at 24 h, 48 h, 72 h, and 96 h was 26.45 mmol/L (Figure 1A), 18.50 mmol/L (Figure 1B), 13.51 mmol/L (Figure 1C), and 10.62 mmol/L (Figure 1D), respectively. The 95% confidence intervals for the LC_50_ of NaHCO_3_ of *M. rosenbergii* at 24 h, 48 h, 72 h, and 96 h were 21.67 to 44.91 mmol/L, 16.74 to 22.10 mmol/L, 12.19 to 14.67 mmol/L, and 8.82 to 11.79 mmol/L, respectively. The safe concentration was 2.71 mmol/L.

### 3.2. Determination of Antioxidant and Immune-Related Indicators

**Figure 2:**
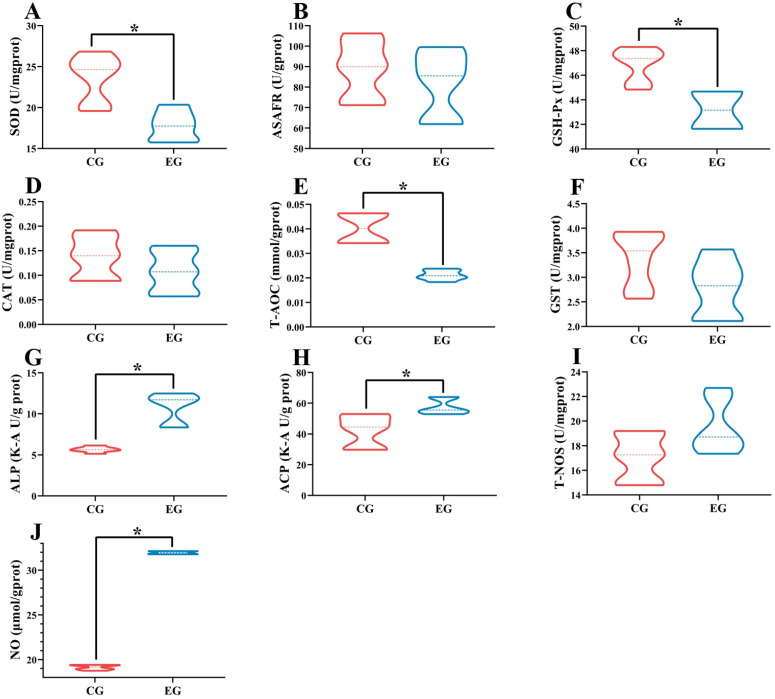
The contents of (**A**) superoxide dismutase (SOD), (**B**) anti-superoxide anion free radical (ASAFR), (**C**) glutathione peroxidase (GSH-Px), (**D**) catalase (CAT), (**E**) total antioxidant capacity (T-AOC), (**F**) glutathione s-transferase (GST), (**G**) alkaline phosphatase (ALP), (**H**) acid phosphatase (ACP), (**I**) nitric oxide synthase (T-NOS), and (**J**) nitric oxide (NO) in the gill tissues of *M. rosenbergii* in the control group and the NaHCO_3_ group. CG, the gill tissues of the control group of *M. rosenbergii*; EG, the gill tissues of the experimental group (NaHCO_3_-exposed group) of *M. rosenbergii*. The asterisk (*) indicates a significant difference between the two groups.

As shown in Figure 2, compared with the control group, antioxidant-related indicators such as the SOD (Figure 2A), GSH-Px (Figure 2C), and T-AOC (Figure 2E) activities were significantly decreased in the NaHCO_3_-exposed group (6.4 mmol/L), and immune-related indicators, including the ALP (Figure 2G), ACP (Figure 2H), and NO (Figure 2J) contents were significantly increased (*p* < 0.05). There were no significant differences in the ASAFR (Figure 2B), CAT (Figure 2D), GST (Figure 2F), and T-NOS (Figure 2I) contents between the control group and the NaHCO3-exposed group (6.4 mmol/L) (*p* > 0.05).

### 3.3. Histological Examination of Gills

**Figure 3:**
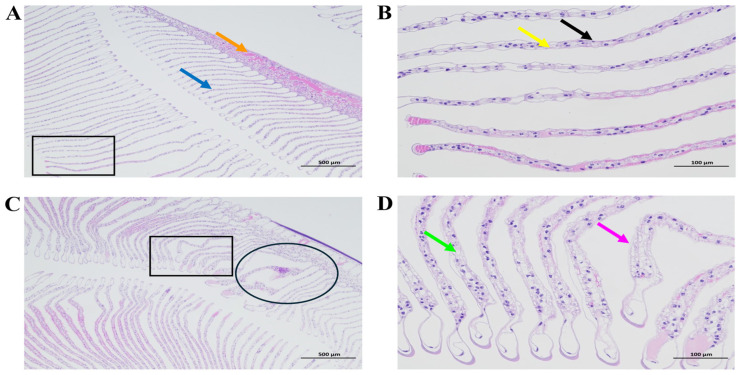
The analysis of gill tissues in *M. rosenbergii* (H&E staining): (**A**,**B**) the control group showing intact tissue architecture; (**C**,**D**) the NaHCO_3_-exposed group caused tissue damage. Magnification: (**A**,**C**) 40×; (**B**,**D**) 200×.

As shown in Figure 3, in the control group, the gill filaments of *M. rosenbergii* were neatly arranged without fractures (blue arrows, Figure 3A). The gill lamellae exhibited intact structures (orange arrows, Figure 3A). The epithelial cells (black arrows, Figure 3B) were regularly aligned, and no significant hyperplasia was observed in the connective tissue (yellow arrows, Figure 3B). No apparent abnormalities were detected. The black square box indicates a magnified field of view (Figure 3A). In the NaHCO_3_-exposed group, the gill filaments of the *M. rosenbergii* exhibited significant thinning accompanied by structural disorganization and tortuous deformities (Figure 3C, circled area). The gill lamellae underwent complete disintegration, rendering their original morphology unrecognizable. The epithelial cells displayed a disordered arrangement, with partial detachment from the gill membrane (purple arrows, Figure 3D). Notably, pronounced vacuolization was observed in the gill cells (green arrows, Figure 3D). The black square box denotes the location of the magnified view (Figure 3C).

### 3.4. Ultrastructure Observation of Gills

**Figure 4:**
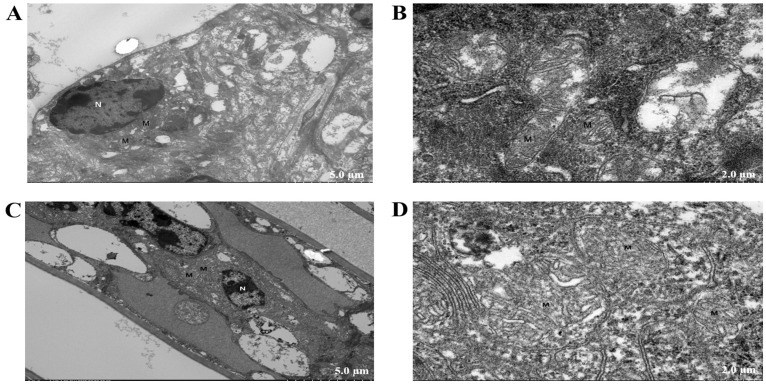
The analysis of gill tissues in *M. rosenbergii* (ultrastructure observation): (**A**,**B**) the control group showing intact tissue architecture; (**C**,**D**) the NaHCO_3_-exposed group caused tissue damage. (**A**,**C**) bar = 5 μm; (**B**,**D**) bar = 2 μm.

As shown in Figure 4, compared to the control group, the gill cells of *M. rosenbergii* in the NaHCO_3_-exposed group exhibited severe edema and disintegration, with extensive dissolution of cell membranes. The nuclei (N) appeared irregularly shaped, accompanied by incomplete cell membranes and dissolution of the cytoplasmic matrix. Mitochondria (M) displayed marked swelling and disintegration, characterized by membrane rupture and extensive dissolution/loss of cristae.

### 3.5. Transcriptomic Library Sequencing Quality

As shown in Table 2, the percentage of high-quality reads was more than 99.58%, that of low-quality reads was less than 0.32%, that of adapter was less than 0.13%, and that of poly A was 0.00%. It was obvious that the sequencing quality was qualified and could be used for subsequent analysis of differentially expressed genes (DEGs).

### 3.6. Reference Alignment

As shown in Table 3, the proportion of unique mapped reads was no less than 84.26%, while the multiple mapped ratio reached a maximum of 6.24%, and the percentage of unmapped reads was no more than 11.51%. The total mapped ratio was at least 88.49%. The high unique mapping rate indicated that the sequencing data were of high quality and suitable for downstream analysis. The unmapped reads were largely composed of sequencing adapters and low-quality bases, which were filtered out.

### 3.7. DEG Analysis

**Figure 5:**
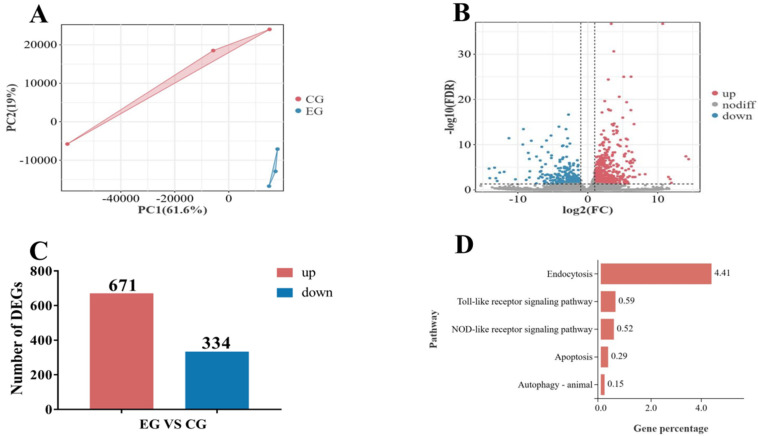
The PCA diagram shows the distribution of data in the principal component space (**A**); the bubble map shows the upregulated and downregulated DEGs (**B**); total DEGs in the gills of *M. rosenbergii* in the NaHCO_3_-exposed group compared to the control group (**C**); the KEGG enrichment analysis results show that some immune-related signaling pathways were involved (**D**).

In this study, principal component analysis (PCA) demonstrated that triplicate samples within each experimental group clustered closely (Figure 5A), indicating minimal intra-group variation and significant inter-group divergence. Volcano plots visualized the distribution of DEGs between comparison groups, with genes positioned toward the extremes of the plot exhibiting greater magnitude of differential expression (Figure 5B). Among the identified DEGs, 671 DEGs were upregulated and 334 DEGs were downregulated (Figure 5C). Kyoto Encyclopedia of Genes and Genomes (KEGG) pathway enrichment analysis revealed the signaling pathways related to immunity and the percentage of DEGs (Figure 5D). The statistical information on immune-related DEGs in the gills of *M. rosenbergii* in the NaHCO_3_-exposed group compared to the control group are shown in Table 4. Log_2_FC stands for log_2_-fold change, a metric that quantifies the difference in gene expression between experimental and control groups on a logarithmic scale. |log_2_FC| > 1 was used as the threshold to identify upregulated DEGs. The *q*-value was the corrected *p*-value, and a *p*-value < 0.05 indicated a statistically significant difference between groups.

### 3.8. qPCR Assay

**Figure 6:**
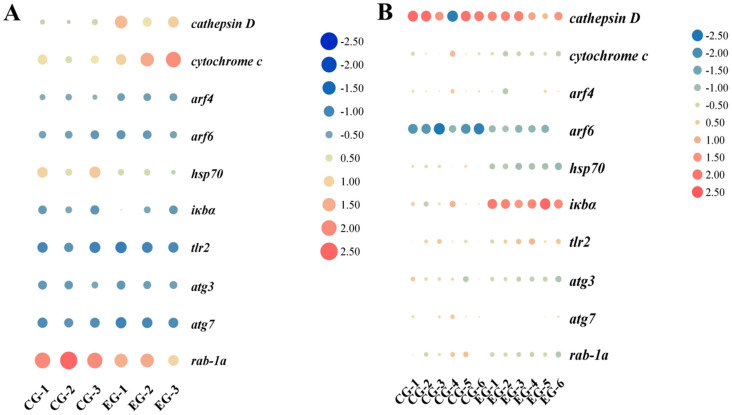

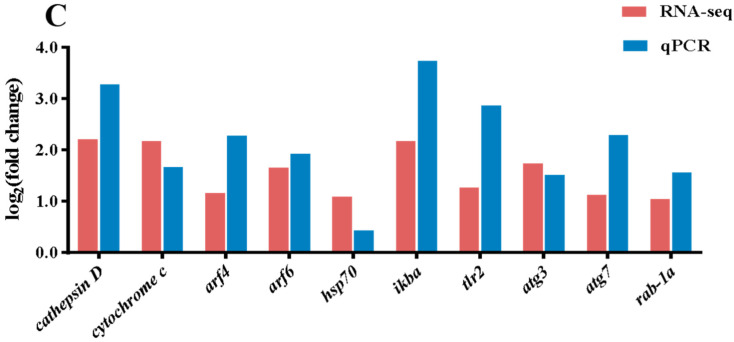
The visualization heat maps of samples and DEGs in RNA-seq and qPCR results; each column in the figure represents a sample and each row represents a gene, and the levels of DEGs in different samples are indicated by different colors (**A**,**B**). CG, the gill tissues of the control group of *M. rosenbergii*; EG, the gill tissues of the experimental group (NaHCO_3_-exposed group) of *M. rosenbergii*. The qPCR results verified the validity of the transcriptome results (**C**). ADP-ribosylation factor 4 (*arf4*), ADP-ribosylation factor 6 (*arf6*), NF-kappa B inhibitor alpha (*iκbα*), heat shock protein 70 (*hsp70*), toll-like receptor 2 (*tlr2*), autophagy 3 (*atg3*), autophagy 7 (*atg7*), ras-related protein Rab-1A (*rab-1a*).

The visualization heat maps of samples and DEGs in the RNA-seq and qPCR results are shown in Figure 6A,B, respectively. The transcriptome results showed that *cathepsin D*, *cytochrome c*, ADP-ribosylation factor 4 (*arf4*), ADP-ribosylation factor 6 (*arf6*), heat shock protein 70 (*hsp70*), NF-kappa B inhibitor alpha (*iκbα*), toll-like receptor 2 (*tlr2*), autophagy 3 (*atg3*), autophagy 7 (*atg7*), and ras-related protein Rab-1A (*rab-1a*) were significantly upregulated in the NaHCO_3_-exposed group compared with the control group. The qPCR results were consistent with the RNA-seq results, confirming the reliability of the RNA-seq results, which could be used for the subsequent analysis (Figure 6C).

### 3.9. Correlation Analysis Between DEGs and Immune Parameters

**Figure 7:**
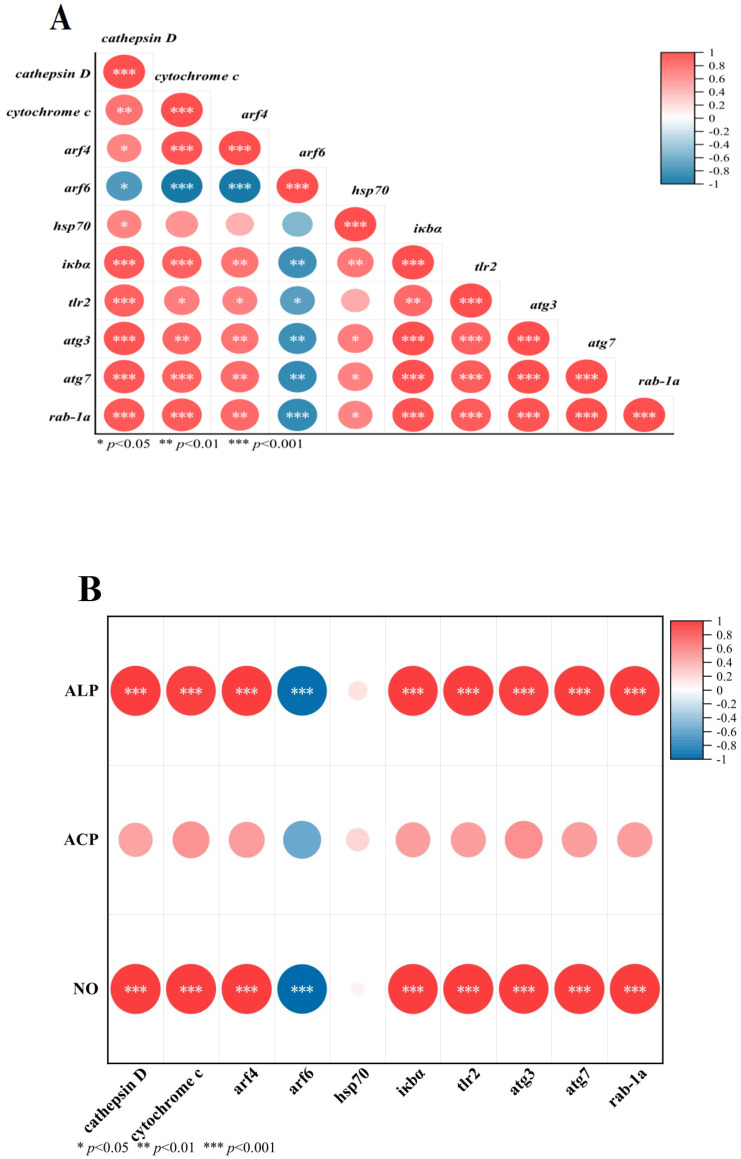
The correlation analysis between DEGs and among DEGs, as well as between DEGs and immune-related enzymes, is shown in (**A**) and (**B**), respectively. *Cathepsin D*, *cytochrome c*, ADP-ribosylation factor 4 (*arf4*), ADP-ribosylation factor 6 (*arf6*), heat shock protein 70 (*hsp70*), NF-kappa B inhibitor alpha (*iκbα*), toll-like receptor 2 (*tlr2*), autophagy 3 (*atg3*), autophagy 7 (*atg7*), ras-related protein Rab-1A (*rab-1a*). Alkaline phosphatase (ALP), acid phosphatase (ACP), nitric oxide (NO).

The correlation analysis among DEGs and between DEGs and immune-related enzymes (ALP, ACP, and NO) is shown in Figure 7A,B, respectively. There was no significant correlation between ACP and DEGs (*p* > 0.05). No significant correlation was found between hsp70 and ALP and NO, but other DEGs showed significant correlations with ALP and NO (*p* < 0.05), among which arf6 showed a significant negative correlation with ALP and NO, and other DEGs were based on a significant positive correlation with ALP and NO.

## 4. Discussion

### 4.1. The Gills of M. rosenbergii Were Damaged Under NaHCO_3_ Stress

In the current study, the transcriptomic analysis revealed a significant upregulation of apoptosis-related genes (*cathepsin D* and *cytochrome c*) in the gill tissues of *M. rosenbergii* under NaHCO_3_ stress. The release and enzymatic activation of *cathepsin D* enable this protease to mediate apoptosis upstream of the caspase cascade [23]. Experimental studies have demonstrated that microinjection of *cathepsin D* triggers *cytochrome c* release from mitochondria, subsequently inducing caspase-dependent apoptosis [24]. These findings suggested that NaHCO_3_ stress may initiate apoptotic pathways in gill tissues. Consistent with this, recent studies on pacific white shrimp have documented similar apoptosis in gill tissues under carbonate/alkaline stress [14,25]. Alkaline stress-induced apoptosis disrupts immune homeostasis and exacerbates tissue damage [26]. Meanwhile, combined with the observation results of transmission electron microscopy, the apoptotic characteristics in the gills of *M. rosenbergii* exposed to NaHCO_3_ conditions, including mitochondrial (M) swelling, disintegration, membrane rupture, and extensive dissolution/loss of cristae, suggested that NaHCO_3_ stress may cause gill tissue damage in *M. rosenbergii* through the apoptotic pathway.

Under mild environmental stress, the upregulation of antioxidant enzyme activity contributes to the efficient clearance of excess reactive oxygen species, but, once the stress exceeds the capacity of the antioxidant system, the defensive function is compromised, leading to a marked decline in the activity of antioxidant enzymes [27,28,29]. SOD and GSH-Px are two of the most critical core enzymes in the cellular antioxidant defense system, and they work synergistically to eliminate harmful reactive oxygen species (ROS) and protect cells from oxidative damage. The current findings indicated that in the NaHCO_3_-exposed group, the levels of SOD and GSH-Px in the gill tissues of *M. rosenbergii* were significantly decreased, along with a notable reduction in T-AOC. This suggested that a NaHCO_3_ concentration of 6.4 mmol/L exceeded the antioxidant defense capacity of *M. rosenbergii*, thereby impairing its antioxidant system. When the production of free radicals exceeds the body’s clearance capacity, the balance is disrupted, leading to oxidative stress which in turn triggers cell apoptosis [30]. Investigations in zebrafish have revealed that oxidative stress subsequently triggers the process of apoptosis [31]. Therefore, we speculated that alkaline stress might disrupt the antioxidant system of *M. rosenbergii*, thereby inducing oxidative stress and ultimately triggering apoptosis; this mechanism still requires further investigation.

### 4.2. Molecular Response Mechanism in Gills of M. rosenbergii Under NaHCO_3_ Stress

The KEGG enrichment results also showed that DEGs were mostly enriched in immune-related metabolic pathways, such as endocytosis, autophagy, and the toll-like receptor signaling pathway. Recent studies have revealed that common carp exposed to high alkalinity stress exhibit significant enrichment of DEGs in the endocytosis pathway [32]. Endocytosis, a critical mechanism for immune defense, is mediated by the activation of ADP-ribosylation factor 4 (*arf4*) and ADP-ribosylation factor 6 (*arf6*) [33,34]. In alignment with these findings, our current research demonstrated a marked upregulation of *arf4* and *arf6* expression in the gill tissues of *M. rosenbergii* under NaHCO_3_ stress. This corroborates earlier work on Chinese white shrimp (*Fenneropenaeus chinensis*), where endocytosis was identified as a key adaptive strategy to counteract hyperalkaline environments [35]. In an acute NaHCO_3_ exposure experiment on oriental river prawn, transcriptomic analysis revealed that both gills and hepatopancreas tissues responded through activation of the endocytosis pathway, underscoring the critical role of endocytosis in combating NaHCO_3_ stress [9,16]. Notably, heat shock protein 70 (*hsp70*) enriched in the endocytosis pathway in the gills was significantly upregulated [16]. These findings aligned with our current results, where *hsp70* expression levels were also markedly elevated in *M. rosenbergii* exposed to NaHCO_3_ conditions. The heat shock protein (HSP) family plays a pivotal role in cellular stress responses, with prior studies establishing *Hsp70* as a key regulator in counteracting environmental stressors [36,37]. For instance, the Chinese mitten crab upregulates *hsp70* expression to mitigate saline/alkaline stress, highlighting its conserved protective function across crustaceans [12]. Building on this, our findings demonstrated that *M. rosenbergii* activated the endocytosis pathway in response to NaHCO_3_ stress by upregulating the expressions of *arf4*, *arf6*, and *hsp70* in gill tissues.

In the current study, the relative expression of NF-kappa B inhibitor alpha (iκbα) was significantly upregulated, with enrichment observed in the toll-like receptor (TLR) signaling pathway. This upregulation of IκBα reflects an adaptive mechanism to counteract adverse environmental stimuli [15]. The TLR pathway, a cornerstone of innate immunity, is pivotal in pathogen recognition and elimination [38,39]. For instance, in the Chinese mitten crab, toll-like receptor 2 (*tlr2*) expression is elevated to mitigate saline/alkaline stress [12]. Our findings align with this, demonstrating a significant increase in *tlr2* expression in the gill tissues of *M. rosenbergii* under NaHCO_3_ stress. Notably, recent studies on crucian carp (*Carassius auratus*) and Pacific whiteleg shrimp have also reported marked enrichment of DEGs in the TLR pathway following carbonate/alkaline stress [40,41].

Autophagy is a dynamic and continuous intracellular degradation process used to remove proteins or damaged organelles due to misfolding and is essential for maintaining homeostasis in the intracellular environment [42,43]. Research indicates that under normal conditions, autophagy operates at a basal level to sustain protein and organelle turnover, but its activity significantly increases in response to diverse physiological and pathological stressors [44]. Enhanced autophagy alleviates ammonia-induced oxidative stress, inflammation, and apoptosis in yellow catfish (*Pelteobagrus fulvidraco*) [45]. In our study, DEGs associated with autophagy—autophagy 3 (*atg3*), autophagy 7 (*atg7*), and ras-related protein Rab-1A (*rab-1a*)—were significantly upregulated in the NaHCO_3_-exposed group. Autophagy 7 [46] and Rab-1A [47] play a crucial role in stress-induced autophagy. These results were consistent with findings in Chinese mitten crab, where carbonate/alkaline stress triggered upregulated expression of autophagy-related mRNAs in hepatopancreatic tissues [15]. Collectively, these data indicated that *M. rosenbergii* activated autophagy pathways to combat NaHCO_3_ stress.

A study has demonstrated that environmental stress significantly increases nitric oxide (NO) levels in *M. rosenbergii* [48]. In the current study, NaHCO_3_ stress individuals showed a marked rise in NO content within gill tissues. Meanwhile, we also found a significant increase in alkaline phosphatase (ALP) and acid phosphatase (ACP) levels in the gill tissues of *M. rosenbergii* under NaHCO_3_ stress. As critical immune parameters in crustaceans, ACP and ALP reflect the host’s immune status under environmental challenges. For instance, during acute carbonate stress, the mud crab (*Scylla paramamosain*) upregulates ACP and ALP activities to counteract external pressures [49]. Similarly, Zhang et al. [10] demonstrated that the Chinese mitten crab enhances ACP and ALP activities to adapt to saline/alkaline stress. Collectively, these findings suggested that *M. rosenbergii* employs an immune strategy by elevating enzymatic activities—including NO, ACP, and ALP—to bolster immunity and mitigate the adverse effects of NaHCO_3_ stress.

The results of KEGG enrichment analysis showed that DEGs were closely related to immune function, and except for *hsp70* and *arf6*, other DEGs had a positive correlation with immune-related enzymes (ALP and NO). The increase in the contents of ALP and NO is an important sign of the improvement of the body’s immune capacity. These results indicated that the immune-related DEGs and immune enzyme activities of *M. rosenbergii* work synergistically to cope with NaHCO_3_ stress, but the specific mechanism by which DEGs interact with ALP and NO still require further research and clarification.

## 5. Conclusions

**Figure 8:**
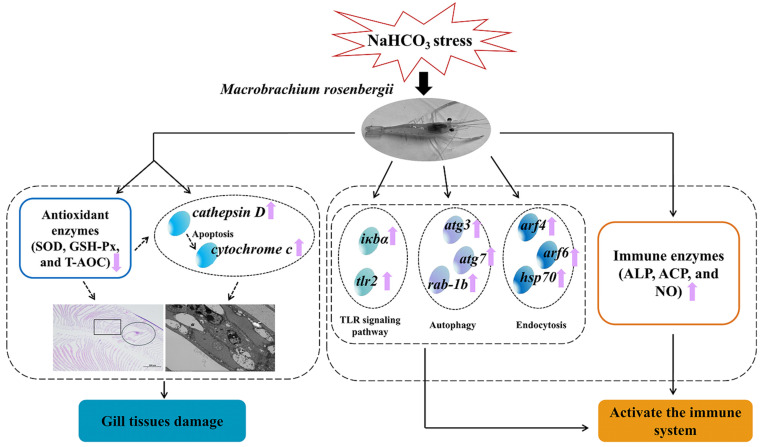
The gill tissue damage and the potential molecular response mechanisms in the gills of *M. rosenbergii* under NaHCO_3_ stress.

Acute NaHCO_3_ stress induced a reduction in antioxidant enzyme activity and gill tissue damage in *M. rosenbergii*. In response to the stress caused by NaHCO_3_, *M. rosenbergii* activated immune-related enzymes (ALP, ACP, and NO) as well as immune-related DEGs involved in endocytosis, autophagy, and the toll-like receptor signaling pathway (Figure 8). The current study revealed the physiological mechanism by which *M. rosenbergii* responds to NaHCO_3_ stress and the damage characteristics it causes. And it provided a crucial scientific basis for utilizing saline/alkaline water resources for shrimp farming, as well as for promoting the ecological restoration and environmental governance of saline/alkaline lands.

## Figures and Tables

**Table 1:** Primer sequences for qPCR.

Gene Name		Sequence	Efficiency (%)	Product Size (bp)	Tm (°C)	Accession Number
beta-actin	F	TCCGTAAGGACCTGTATGCC	109.4	198	56.70	
	R	TCGGGAGGTGCGATGATTTT			56.79	
*cathepsin D*	F	CTGAGGATGAAGGTGTTGAT	104.5	231	51.48	AMQ98967.1
	R	CTGAGGAGGAGTGCCAAT			54.28	
*cytochrome c*	F	ACAGATGCTAACAAGTCCAA	107.9	155	50.69	XP_063881551.1
	R	TCCTCAAGGTAGGCTATCAA			51.90	
*arf4*	F	GTCTTGATGCTGCTGGTA	103.4	116	51.79	XP_064122923.1
	R	GCTGATGTTCTTGTATTCCA			49.32	
*arf6*	F	ACGAAGCAAGGCAAGAAT	109.8	198	51.60	KAK7082885.1
	R	ATAGTCCATCACCTGTTGTT			50.21	
*hsp70*	F	AGCAGACTCAGACATTCAC	108.5	199	51.53	AAS45710.1
	R	GACACATTCAGGATACCATTG			50.52	
*iκbα*	F	ACCTCACTAACGCTACGA	98.4	252	52.38	AET34918.1
	R	ACACTGCCAGATGTAACG			52.02	
*tlr2*	F	CAACGGCAATCCTGACTT	91.5	219	52.65	XP_064078749.1
	R	CGACGAATCACATTAGAAGAG			50.18	
*atg3*	F	ACGATGATGATGACGATGA	93	259	50.11	QCX35196.1
	R	GAGAGTGGCTGACGATTC			52.84	
*atg7*	F	GTCATCGTCCTGGTCTTG	108.1	118	52.81	QEG53818.1
	R	GGCATTCCACAACTGAGA			52.05	
*rab-1a*	F	GCTCACGGCATCATAGTT	102.8	179	52.18	XP_064110114.1
	R	GCATATTCCTTGGCTGTCT			52.04	

Note: ADP-ribosylation factor 4 (*arf4*), ADP-ribosylation factor 6 (*arf6*), NF-kappa B inhibitor alpha (*iκbα*), heat shock protein 70 (*hsp70*), toll-like receptor 2 (*tlr2*), autophagy 3 (*atg3*), autophagy 7 (*atg7*), ras-related protein Rab-1A (*rab-1a*).

**Table 2:** Quality assessment of transcriptome library sequencing.

Sample	Raw Data	Clean Data (%)	Adapter (%)	Low Quality (%)	Poly A (%)
CG-1	41,185,602	41,064,878 (99.71%)	6832 (0.02%)	113,234 (0.27%)	0 (0.00%)
CG-2	42,137,954	42,002,718 (99.68%)	39,714 (0.09%)	95,212 (0.23%)	0 (0.00%)
CG-3	41,255,780	41,122,472 (99.68%)	11,752 (0.03%)	121,042 (0.29%)	0 (0.00%)
EG-1	36,415,096	36,303,148 (99.69%)	5332 (0.01%)	106,368 (0.29%)	0 (0.00%)
EG-2	45,237,474	45,092,932 (99.68%)	9120 (0.02%)	133,298 (0.29%)	0 (0.00%)
EG-3	43,778,564	43,593,874 (99.58%)	50,534 (0.12%)	134,156 (0.31%)	0 (0.00%)

Note: Raw Data, number of raw reads; Clean Data, number and percentage of high-quality reads (based on raw reads); Adapter, number of reads containing adapter and percentage (based on raw reads); Low Quality, low-quality reads, that is, the number and percentage (based on raw reads) of reads with more than 50% base mass value Q ≤ 20 in single-ended read; Poly A, number and percentage of poly A reads (based on raw reads). CG, the gill tissues of the control group of *M. rosenbergii*; EG, the gill tissues of the experimental group (NaHCO_3_-exposed group) of *M. rosenbergii*.

**Table 3:** Mapping statistics against reference genome.

Sample	Total	Unmapped (%)	Unique Mapped (%)	Multiple Mapped (%)	Total Mapped (%)
CG-1	36,311,924	4,120,293 (11.35%)	30,596,384 (84.26%)	1,595,247 (4.39%)	32,191,631 (88.65%)
CG-2	32,383,102	2,663,419 (8.22%)	27,698,895 (85.54%)	2,020,788 (6.24%)	29,719,683 (91.78%)
CG-3	37,078,672	4,267,183 (11.51%)	31,489,881 (84.93%)	1,321,608 (3.56%)	32,811,489 (88.49%)
EG-1	32,991,962	2,993,126 (9.07%)	28,867,018 (87.50%)	1,131,818 (3.43%)	29,998,836 (90.93%)
EG-2	40,401,422	3,395,288 (8.40%)	35,561,175 (88.02%)	1,444,959 (3.58%)	37,006,134 (91.60%)
EG-3	38,749,590	3,022,772 (7.80%)	34,284,339 (88.48%)	1,442,479 (3.72%)	35,726,818 (92.20%)

Note: Unmapped (%), number and percentage of reads not aligned to the reference genome; Unique Mapped (%), number and percentage of reads uniquely aligned to the reference genome; Multiple Mapped (%), number and percentage of reads aligned to multiple locations in the reference genome; Total Mapped (%), number and percentage of all reads successfully aligned to the reference genome. CG, the gill tissues of the control group of *M. rosenbergii*; EG, the gill tissues of the experimental group (NaHCO_3_-exposed group) of *M. rosenbergii*.

**Table 4:** Information statistics of immune-related DEGs in the gills of *M. rosenbergii* in the NaHCO_3_-exposed group compared to the control group.

DEGs	Signaling Pathway Name	|log_2_FC|	*q* Value
*cathepsin D*	Apoptosis	2.213	0.000
*cytochrome c*		2.174	0.000
*arf4*	Endocytosis	1.161	0.004
*arf6*		1.650	0.000
*hsp70*		1.093	0.003
*iκbα*	Toll-like receptor signaling pathway	2.177	0.000
*tlr2*		1.276	0.034
*atg3*	Autophagy	1.738	0.008
*atg7*		1.131	0.033
*rab-1a*		1.043	0.002

Note: ADP-ribosylation factor 4 (*arf4*), ADP-ribosylation factor 6 (*arf6*), NF-kappa B inhibitor alpha (*iκbα*), heat shock protein 70 (*hsp70*), toll-like receptor 2 (*tlr2*), autophagy 3 (*atg3*), autophagy 7 (*atg7*), ras-related protein Rab-1A (*rab-1a*).

## Data Availability

The raw data of the present study have been submitted to the NCBI with the accession number PRJNA1337614. All other data are contained within the manuscript.
